# Site-Specific Glyco-Tagging of Native Proteins for the Development of Biologicals

**DOI:** 10.1021/jacs.4c11091

**Published:** 2024-12-09

**Authors:** Ana Gimeno, Anna M. Ehlers, Sandra Delgado, Jan-Willem H. Langenbach, Leendert J. van den Bos, John A.W. Kruijtzer, Bruno G.A. Guigas, Geert-Jan Boons

**Affiliations:** 1 Chemical Biology and Drug Discovery, Utrecht Institute for Pharmaceutical Sciences, and Bijvoet Center for Biomolecular Research, Utrecht University, Utrecht, CG 3584, The Netherlands; 2 CIC bioGUNE, Basque Research & Technology Alliance (BRTA), Bizkaia Technology Park, Building 800, Derio 48160, Bizkaia Spain; 3 EnzyTag BV, Daelderweg 9, Nuth, HK NL-6361, The Netherlands; 4 Leiden University Center of Infectious Diseases, Leiden University Medical Center, Leiden, ZA 2333, The Netherlands; 5 Complex Carbohydrate Research Center, University of Georgia, Athens, Georgia 30602, United States; 6 Department of Chemistry, University of Georgia, Athens, Georgia 30602, United States

## Abstract

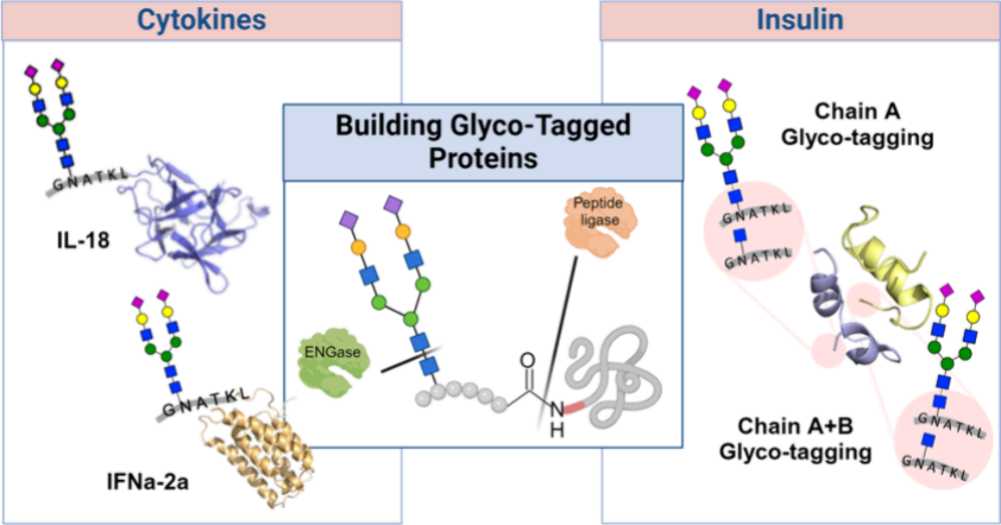

Glycosylation is an attractive approach to enhance biological properties of pharmaceutical proteins; however, the precise installation of glycans for structure–function studies remains challenging. Here, we describe a chemoenzymatic methodology for glyco-tagging of proteins by peptidoligase catalyzed modification of the *N*-terminus of a protein with a synthetic glycopeptide ester having an *N*-acetyl-glucosamine (GlcNAc) moiety to generate an *N*-GlcNAc modified protein. The GlcNAc moiety can be elaborated into complex glycans by *trans*-glycosylation using well-defined sugar oxazolines and mutant forms of endo β-*N*-acetylglucosaminidases (ENGases). The glyco-tagging methodology makes it possible to modify *on-demand* therapeutic proteins, including heterologous proteins expressed in *E. coli*, with diverse glycan structures. As a proof of principle, the *N*-terminus of interleukin (IL)-18 and interferon (IFN)α-2a was modified by a glycopeptide harboring a complex *N*-glycan without compromising biological potencies. The glyco-tagging methodology was also used to prepare several glycosylated insulin variants that exhibit reduced oligomerization, aggregation, and fibrillization yet maintained cell signaling properties, which are attractive for the development of insulins with improved shelf-lives. It was found that by employing different peptidoligases, it is possible to modify either the A or both chains of human insulin.

## Introduction

Biologicals are a fast-growing class of pharmaceuticals that accounted for approximately one-third of drugs approved by the FDA in 2022.^1,2^ However, native peptides and proteins often exhibit poor pharmacokinetic (PK) profiles that undergo rapid proteolytic degradation or clearance. PEGylation is a widely applied strategy to improve PK profiles and biological storability of biologics, and to date, 38 PEGylated therapeutics have been approved by the FDA, including growth factors, erythropoietin (EPO), interferons, and coagulation factors.^3^*N*-Terminal site-specific PEGylation is attractive and provides therapeutics with improved PK profiles with minimal interference of the protein’s secondary structure and biological activity.^4^ Safety concerns such as PEG hypersensitivity, immunogenicity and bioaccumulation have, however, been noted,^5−7^ and other pegylated products have failed clinical testing such as PEGylated insulin (Lispro) due to hepatic toxicity.^8^ As an alternative strategy, glycosylation, which is a common post-translational modification,^9^ can be exploited to enhance properties of biopharmaceuticals. In fact, the incorporation of carbohydrate polymers such as dextran or polysialylation has received considerable attention.^10^ Glycosylation can be an important determinant of PK properties, cellular distributions, and biological activities of therapeutic glycoproteins.^11,12^ It can improve the solubility, thereby preventing aggregation and elimination. Glycosylation can also increase the stability of a protein by preventing proteolytic degradation. It can mask potential antigenic epitopes and thus prevent unwanted immunological reactions. Glycosylation also increases the overall size of a protein, thereby reducing clearance and extending the half-life of a protein. By fine-tuning the structure of a glycan moiety of a glycoprotein, specific cell types can be targeted and hepatic clearance controlled. Glycosylated variants of proteins such as human growth hormones,^13^ insulin,^14−19^ and glucagon-like peptide-1^20,21^ exhibit improved therapeutic profiles compared to their nonglycosylated counterparts, without compromising functional activities. *N*-Terminal glycosylation of insulin has provided variants with prolonged glucose-lowering effects, improved PK and PD, and reduced fibrillation; however, their synthesis involves complex and/or inefficient approaches.^14,15,17−19^ Similarly, fusion proteins bearing an additional glycosylated peptide domain have also been described with improved PK profiles.^22,23^ For example, corifollitropin alfa (Elonva) is a recombinant follicle-stimulating hormone (FSH) analogue that is composed of the β-subunit of FSH fused to the C-terminal peptide of the human chorionic gonadotropin (hCG) β-subunit containing four *O*-linked glycosylation sites. This chimeric protein has a 4-fold increase in elimination half-life and enhanced *in vivo* bioactivity compared to wild-type FSH.^24−26^ Thus, the assembly of fusion proteins tagged with a peptide sequence containing one or more glycosylation sites is an attractive strategy to improve the properties of biologicals.

Despite advances, controlled modification of proteins with glycans remains challenging, thereby complicating structure–function studies. Glycan structures are not precisely defined at the genetic level, and as a result, eukaryotic expression systems generally provide mixtures of different glycoforms.^27^ This hurdle may be overcome by synthetic or semisynthetic approaches,^28−33^ although current methodologies to modify native proteins with specific glycans often lack selectivity, leading to heterogeneity.^34^

**Figure 1:**
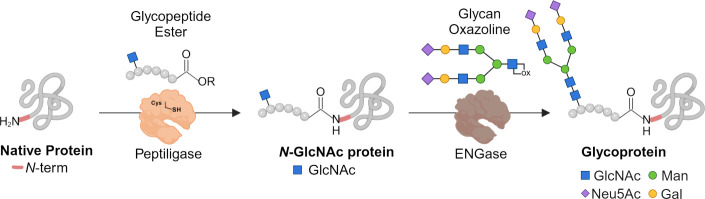
Chemoenzymatic strategy for specific glyco-tagging of native proteins.

Here, we describe a chemoenzymatic methodology that makes it possible to modify the *N*-terminus of native proteins with well-defined glycopeptide tags without introducing non-natural modifications.^35^ It leverages peptidoligases^36^ and transglycosidases^37−39^ for the controlled modification of the *N*-terminus of a protein with an *N*-glycosylated sequon. Specifically, the *N*-terminus of a protein is ligated with a synthetic glycopeptide ester having an *N*-acetyl-glucosamine (GlcNAc) moiety using a peptidoligase^36,40^ to generate a *N*-GlcNAc modified protein (Figure 1). Next, the GlcNAc moiety of the resulting ligation product can be elaborated into complex glycans by *trans*-glycosylation using mutant forms of endo β-*N*-acetylglucosaminidases (ENGases).^37−39^ The glyco-tagging methodology described here enables modifying *on-demand* therapeutic proteins, including proteins expressed in *E. coli*, with diverse glycan structures. As a proof of principle, we employed the approach to modify the *N*-terminus of cytokines interleukin-18 (IL-18) and interferon alpha-2a (IFNα-2a) by a glycopeptide harboring a complex *N*-glycan and demonstrated that the modification does not affect biological potencies. The methodology was also employed to prepare several glycosylated insulin variants that exhibit reduced oligomerization, aggregation, and fibrillization yet maintain cell signaling properties. By employing different peptidoligases, it was possible to modify either the A or both chains of human insulin.

## Results and Discussion

### Chemical Synthesis of a Glycopeptide Ester and Model Ligation

Pioneering research by Weeks and Wells^36^ has shown that rationally engineered forms of subtilisin from *B. subtilis* are devoid of hydrolytic activity and can make peptide bonds at the *N*-terminal α-amine of a peptide by employing an appropriate peptide ester. The active sites of these so-called peptidoligases have been further engineered to accommodate a wide range of amino acid side chains at the C- and N-terminal ligation junction, resulting in enzymes with broader substrate specificity.^40^

**Figure 2:**
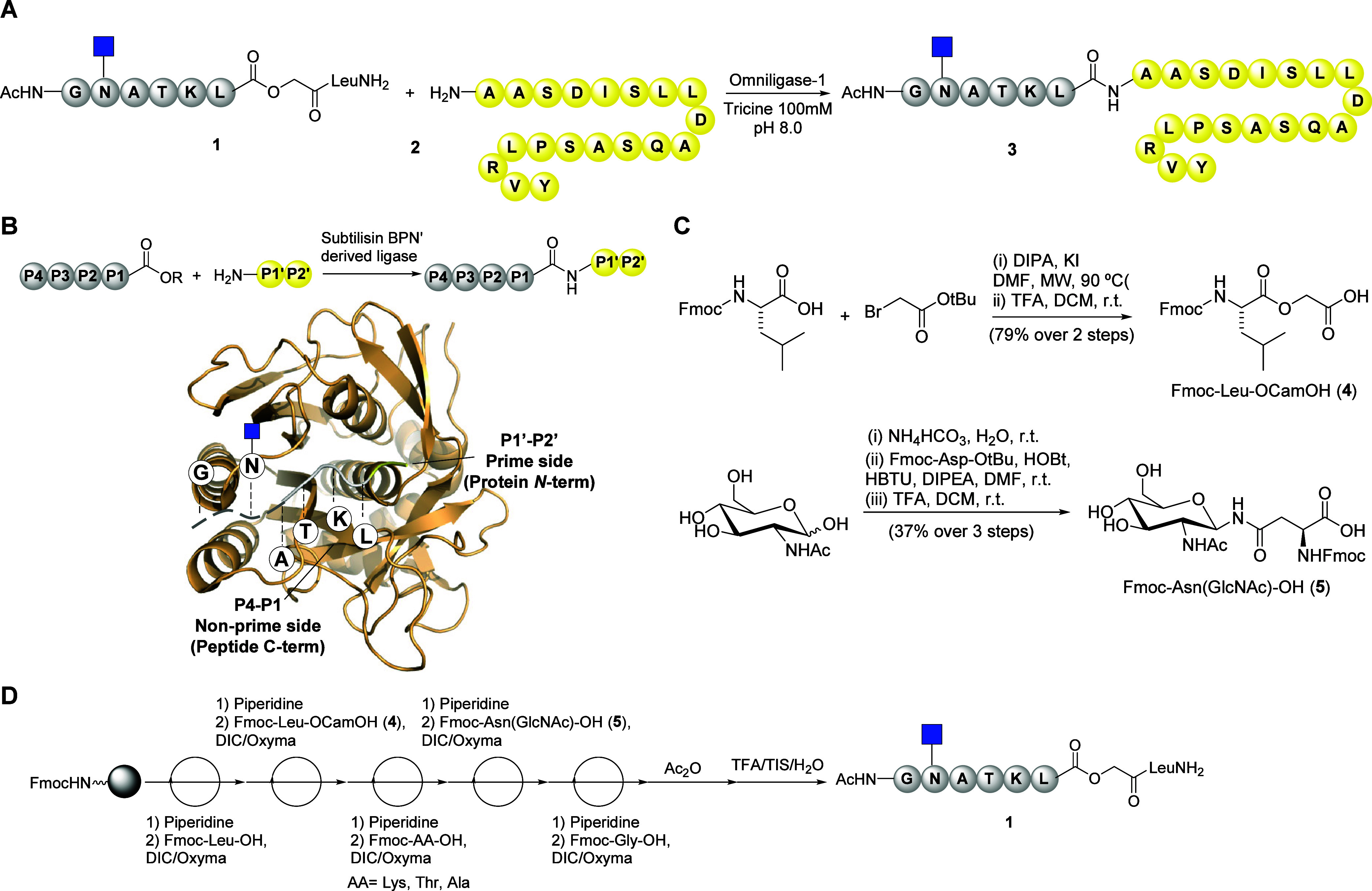
Synthesis and reactivity assays of glycopeptide ester **1**. (A) Reaction between glycopeptide **1** and an *N*-terminal dialanine model peptide **2**. The ligation product **3** was obtained in a 74% isolated yield referred to as glycopeptide **1**, used as the limiting reagent. Reaction was performed using a 2:1 ratio of peptide **2** to glycopeptide **1** in the presence of 1.5 mol % enzyme loading. (B) Peptide ligation catalyzed by subtilisin-derived ligases. Below: structure of subtilisin BPN′ (PDB 1SBN) with a schematic representation of P4–P1 and P1′–P2′ positions of the acyl donor and acyl acceptor fragments, respectively, which are important for successful peptide ligation. (C) Chemical synthesis of building blocks **4** and **5**. (D) Solid-phase peptide synthesis of glycopeptide ester **1**.

Although peptidoligases have found various applications,^36^ very few reports deal with the *N*-terminal modification of proteins.^41,42^ Furthermore, except for the early investigations by Wong and co-workers dealing with subtilisin-catalyzed glycopeptide synthesis,^43,44^ there are no reports describing the synthesis of unprotected glycosylated peptide esters suitable for peptidoligase-mediated preparation of glycoproteins. We synthesized a glycopeptide ester having an *N*-glycosylation site that was employed for a ligation with a model peptide substrate using omniligase-1, which is a readily available and well-characterized peptidoligase.^45,46^ As a glycopeptide ester, we selected compound **1**, which was ligated with peptide **2** to give glycopeptide **3** (Figure 2A). The P4–P1 residues of the acyl donor are important for ligation efficiency (Figure 2B), and therefore, **1** incorporates amino acids at these positions that are preferred by omniligase-1.^47^ To prevent the glycosylation site of the glycopeptide ester from interfering with the activity of the peptidoligase, it was positioned outside the binding pocket of the enzyme. Furthermore, it contains a typical amino acid sequence for *N*-glycosylation (N-X-S/T) to provide a natural *N*-glycan sequon. In particular, the N-A-T sequence was selected because of its high abundance in natural glycoproteins.^48^ To avoid any further reactivity of glycopeptide **1**, the *N*-terminal α-amine was capped by acetylation. The *N*-terminal amino acid sequence of the acyl acceptor is also important for the ligation efficiency. Omniligase-1 prefers small apolar amino acids at positions P1′ and P2′, and therefore, the *N*-terminus of **2** has an Ala-Ala sequence at these positions (Figure 2B).

The carboxyamidomethyl ester (Cam) linkage of **1** was installed using Fmoc-Leu-OCH_2_COOH (**4**) as a building block (Figure 2C), which could readily be prepared by condensation of Fmoc-protected leucine with *tert*-butyl bromoacetate followed by the removal of the *t*-butyl ester using TFA in DCM (79% overall yield). The Cam ester was selected because it is an attractive acyl donor substrate for peptidoligase-catalyzed reactions due to the structural similarity to natural protease substrates and a good balance between reactivity and stability.^49,50^ The glycosylated amino acid Fmoc-Asn(GlcNAc)-OH (**5**) was employed to install the carbohydrate moiety of **1**, which was prepared by Kochetkov amination of *N*-acetyl-glucosamine followed by coupling of the resulting α-glycosylamine with *N*-α-Fmoc-protected l-aspartic acid-*tert*-butyl ester and then by treatment with TFA/DCM to remove the *t*-butyl ester (Figure 2C).^51^ The GlcNAc-containing glycopeptide ester **1** was synthesized on a Rink Amide AM LL resin using a CEM Liberty 12-channel automated microwave peptide synthesizer employing a standard Fmoc-solid-phase peptide synthesis protocol (Figure 2D). The glycopeptide ester was cleaved from the resin with the simultaneous removal of the side chain protecting groups of the amino acids by treatment with TFA/TIS/water and then purified by HPLC using a C18 reverse column. Mass spectrometry and NMR confirmed the structural integrity of the compound. Ligation of glycopeptide ester **1** with peptide **2** in the presence of omniligase-1 proceeded smoothly in 100 mM tricine at pH 8.0 to provide the corresponding glycopeptide **3** in a 74% yield after purification by C18 reverse phase HPLC (Figure 2A).^52^ A byproduct was also isolated in which the ester of **1** had been hydrolyzed.

### Ligation of Glycosylated Peptide Esters with Proteins

Encouraged by the successful synthesis of glycopeptide **3** by peptide ligation, attention was focused on the modification of proteins with a glycopeptide tag. First, the ligation of glycopeptide ester **1** with the carbohydrate binding domain of human Galectin-3 (*h*Gal3-CRD), which can easily be expressed in *E. coli*,^53^ was investigated. The reaction was conducted at 100–150 μM protein concentration with 1 mol % omniligase-1 and analyzed by mass spectrometry after 2 h (Table S5 for additional details), which indicated a relatively low conversion of ∼20%. *h*Gal3-CRD has the nonpolar residues Met-Leu at the *N*-terminus, which is suboptimal for omniligase-1, at least under the employed reaction conditions. Therefore, we investigated thymoligase as peptide ligase, which was developed to accept negatively charged amino acids at position P1′.^54^ Interestingly, the use of this ligase resulted in an improved conversion of ∼50%, demonstrating its compatibility with other amino acids, including those with amphipathic characteristics such as methionine.

Next, attention was focused on glyco-tagging of the biomedically important cytokines IL-18 and IFNα-2a. Cytokines are small signaling proteins that can modulate processes such as inflammation and the immune response. They are emerging as attractive therapeutics for various immune-related diseases; however, their short half-lives limit their application, and methodologies are needed to increase the stability of these proteins.^55^ IL-18 is experiencing a renascence in cancer therapy by enhancing IFN-γ production by tumor-infiltrating T cells,^56^ and IFNα-2a is an established therapeutic for the treatment of hepatitis B and C (brand name of pegylated form: Pegasys) due to its antiproliferative capacity on virus-infected immune cells.^57,58^

**Figure 3:**
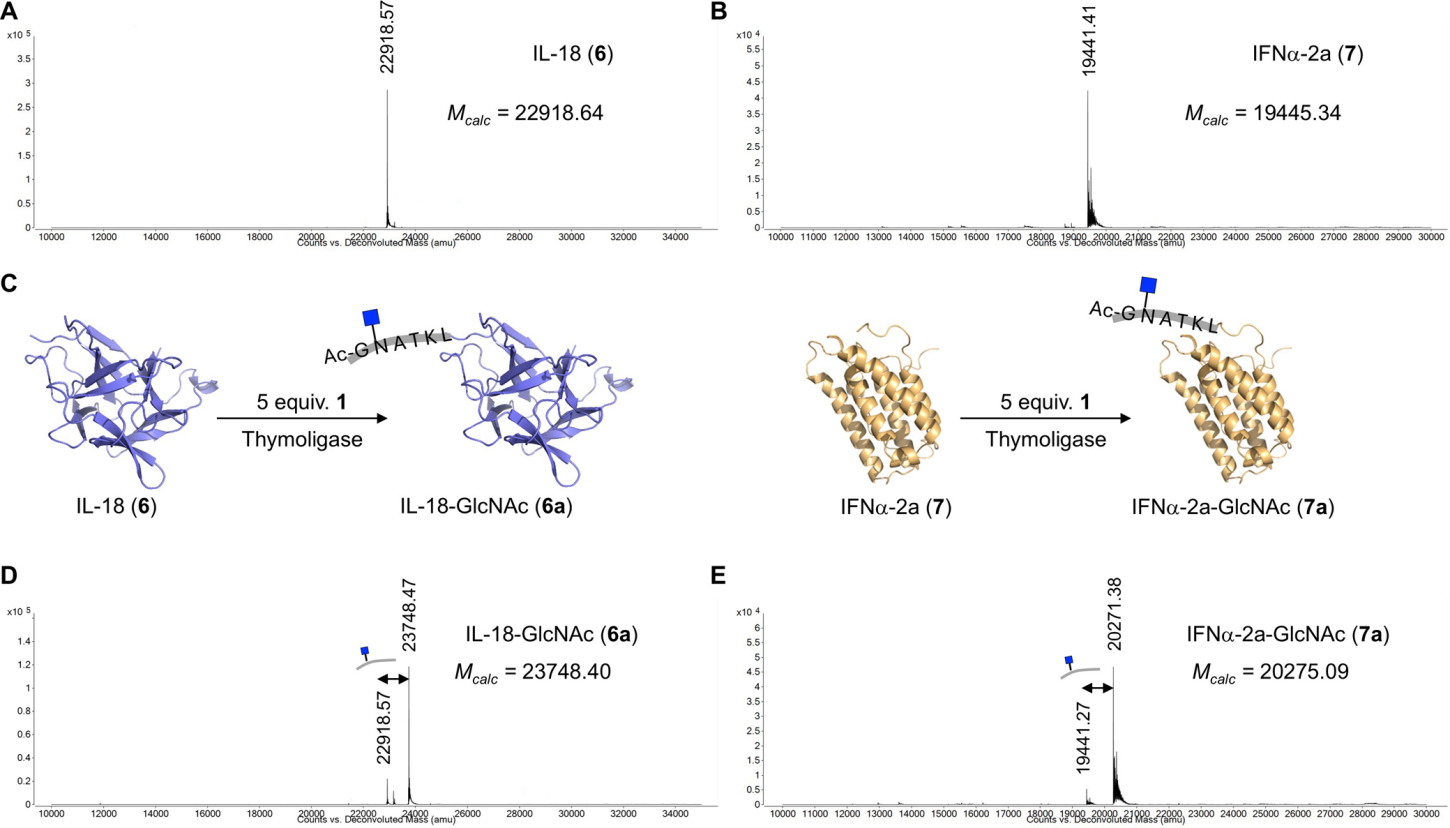
Chemoenzymatic synthesis of IL-18 and IFNα-2a glycovariants **6a** and **7a**. (A, B) Deconvoluted mass spectra of IL-18 (**6**) and IFNα-2a (**7**) recombinantly expressed using *E. coli*. (C) Reaction of glycopeptide **1** with IL-18 (**6**) or IFNα-2a (**7**) provided homogeneous *N*-acetyl glucosamine modified proteins **6a** and **7a**. (D, E) Deconvoluted mass spectra of IL18-GlcNAc (**6a**) and IFNα-2a-GlcNAc (**7a**).

Both proteins were recombinantly expressed in *E. coli*(59,60) as soluble entities (Figures S6 and S7). A TEV (tobacco etch virus) protease cleavage site (ENLYFQ/G) was introduced into the respective synthetic gene constructs to expose amino acids other than methionine, which serves as the initiation translating residue in *E. coli*, at the *N*-terminus of the proteins. After TEV cleavage, the IL-18 and IFNα-2a sequences incorporate *N*-terminal Gly, whose small and hydrophobic character would enhance the efficiency of ligation.^47^ In the case of IFNα-2a, whose natural sequence starts with a Cys involved in a disulfide bond, an additional apolar residue was included so that the final protein contained a Gly-Phe moiety at the *N*-terminus. The integrity of the expressed proteins was confirmed by intact protein MS after TEV cleavage and purification by affinity chromatography (Figure 3A,B). The ligation between IL-18 (**6**) or IFNα-2a (**7**) and glycopeptide ester **1** (5 equiv) was performed with thymoligase and provided the corresponding GlcNAc variants **6a** and **7a**, respectively (Figure 3C). The employed thymoligase contained a His tag and could therefore be removed by Ni-NTA affinity chromatography. Analysis of the protein fractions by intact protein mass spectrometry showed predominant species corresponding to the ligation of glycopeptide **1** to IL-18 and IFNα-2a with peaks with *m*/*z* at 23748.47 and 20271.38 Da, respectively (Figure 3D,E). A small amount of unmodified protein was detected in each case.

### *N*-Glycosylation of Glyco-Tagged Proteins

Because the favorable impact of sialylation on serum half-life of biotherapeutics is well-known,^61−63^ we focused on modifying GlcNAc-containing IL-18 (**6a**) and IFNα-2a (**7a**) with a complex-type biantennary *N*-sialoglycan. As a convergent approach for *N*-glycoprotein assembly, we explored glycosylation by treatment with endoglycosidase (ENGase) mutants in combination with sugar oxazolines as activated donor substrates.^64,65^ ENGases are endoglycosidases that cleave *N*-glycans from glycoproteins by hydrolyzing the glycosidic bond of the chitobiose core. Several ENGases have been identified that possess transglycosylation activity and can transfer a released *N*-glycan to a GlcNAc acceptor to form a new glycosidic linkage.^64,66,67^ Subsequently, it was found that synthetic glycan oxazolines, which are mimics of the oxazolinium ion intermediate,^68−70^ are transferred more efficiently.^71,72^ To address the problem of product hydrolysis, mutants of ENGases have been developed that lack hydrolytic activity but can still use the activated sugar oxazolines for transglycosylation, thereby making it possible to prepare well-defined glycopeptides and glycoproteins.^37,38,64^

**Figure 4:**
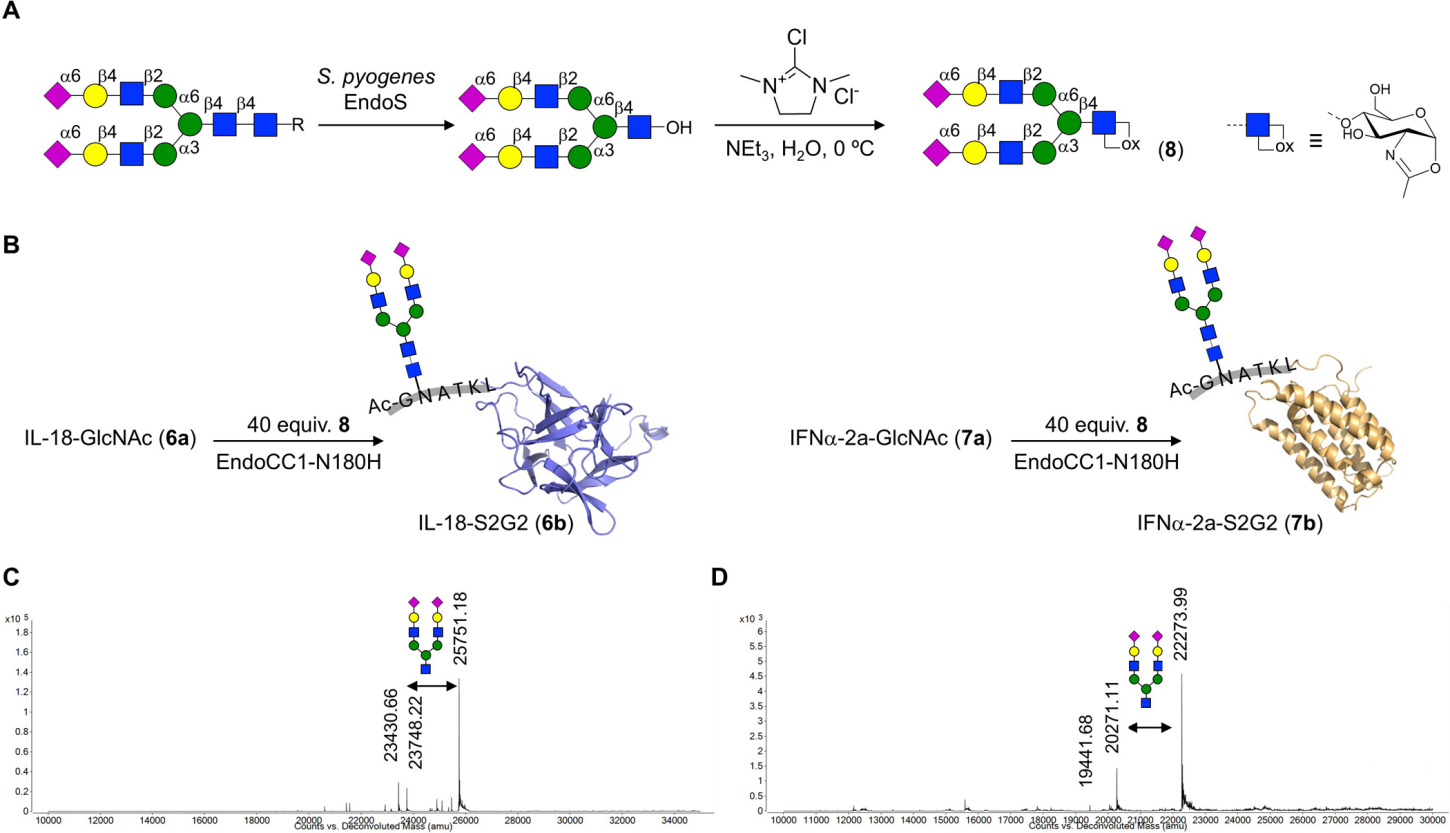
Chemoenzymatic protein remodeling to afford sialylated IL-18 and IFNα-2a glycovariants **6b** and **7b**. (A) Conversion of sialoglycopeptide (SGP) isolated from egg yolk powder into oxazoline **8**. (B) Protein-glycopeptide conjugates **6a** and **7a** were transformed into complex-type glycosylated variants **6b** and **7b** by reaction with oxazoline **8** in the presence of EndoCC1-N180H. (C, D) Deconvoluted mass spectra of glycovariants (**6b**) and (**7b**).

We selected a mutant ENGase from *Coprinopsis cinerea* (EndoCC1-N180H)^73^ to modify **6a** and **7a** with oxazoline **8** to give glycoproteins **6b** and **7b**, respectively. This mutant ENGase was selected because it displays a similar specificity as EndoM to transfer biantennary sialoglycans to proteins, is more easily expressed, and is more thermostable.^74^ Disialoglycan oxazoline **8** was prepared from the corresponding sialoglycopeptide isolated from egg yolk powder^75^ by treatment with EndoS to cleave the glycosidic bond of the chitobiose core followed by reaction with 2-chloro-1,3-dimethylimidazolium chloride (DMC)^76^ in the presence of triethylamine to convert the reducing *N*-acetyl-glucosamine moiety into an oxazoline (Figure 4A).^70^ The transglycosylation was carried out using 40 equiv of **8** and 0.5 mol % EndoCC1-N180H in 50 mM Tris (pH 7.5) at room temperature for 1 h. If starting protein remained, an additional portion of oxazoline **8** was added, and incubation was continued for 30 min (Figure 4B). Analysis of the reaction mixture by intact protein MS indicated conversions for IL-18-GlcNAc (**6a**) and IFNα-2a-GlcNAc (**7a**) of ∼90 and ∼51%, respectively. Disialylated IL-18 (IL-18-S2G2, **6b**) was purified by Strep-tag affinity chromatography (Figure 4C for intact MS data). In the case of IFNα-2a, no affinity tag was incorporated in the protein construct, and therefore, EndoCC1-N180H, which has a His_8_-tag, was removed by Ni-NTA affinity chromatography, and the resulting protein was subjected to concanavalin A affinity chromatography to afford glycosylated IFNα-2a (**7b**). To further remove incomplete modified products, the protein was subjected to size exclusion chromatography using a ReproSil 125 SEC column, which gave homogeneous **7b** (Figure 4D). These results indicate that the combination of site-selective GlcNAc-peptide ligation and chemoenzymatic glycan elaboration enables the installation of well-defined complex glycans within a target protein. The results demonstrated the feasibility of the glyco-tagging methodology to transform bacterially expressed proteins into well-defined glycosylated variants.

### The Effect of Glycosylation on Biological Activities of Glyco-Tagged Cytokines

**Figure 5:**
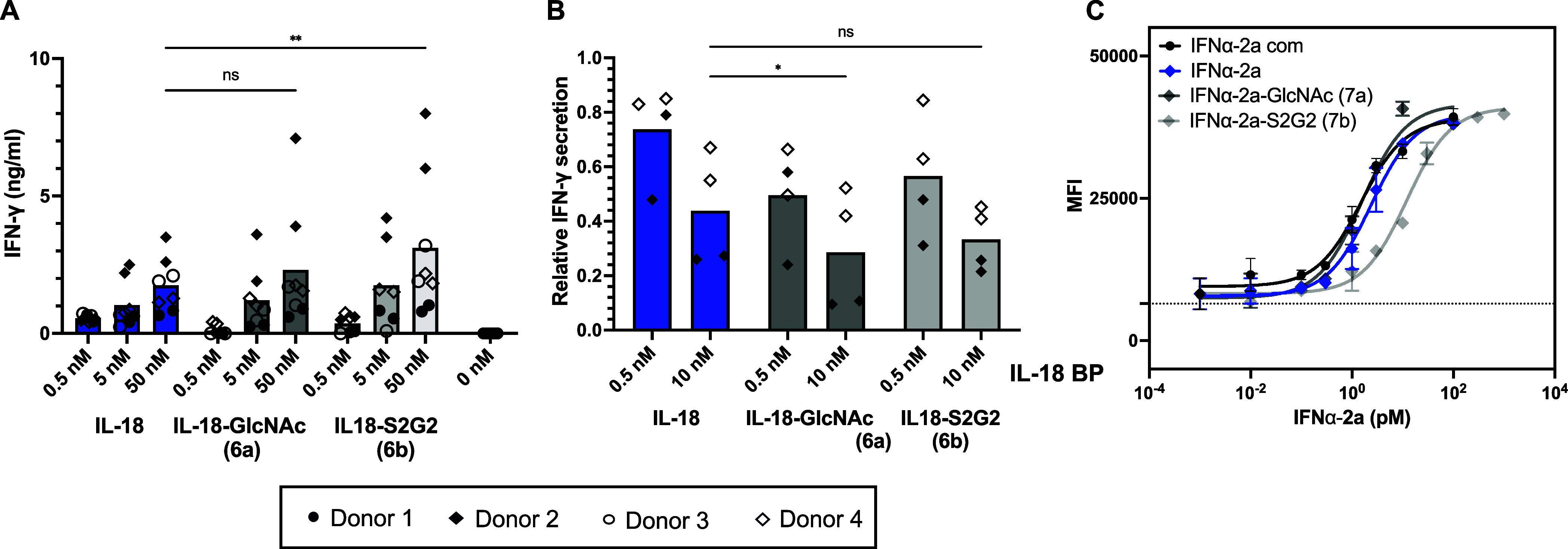
Biological activities of glycan-tagged variants of IL-18 (**6a**, **6b**) and IFNα-2a (**7a**, **7b**). (A) Human PBMCs (donors *n* = 4, biological duplicates) were stimulated with IL-18 variants (0.5, 5.0, and 50 nM) in the presence of IL-12 (10 ng/mL), and IFN-γ release was monitored. Data are represented as means combined with each replicate, and statistical significance was tested with nonparametric one-way ANOVA (Friedman test); **p < 0.01. Inhibition of IL-18 (10 nM) induced IFN-γ release by the decoy protein IL-18 BP (0.5 and 10 nM) is shown as biological replicates (donors *n* = 2) and is normalized to the average release of each glyco-tagged variant at 10 nM in the absence of IL-18 BP. Nonparametric one-way ANOVA (Friedman test) was used for statistical analysis; *p < 0.05 C) CFSE-labeled Daudi cells were stimulated with half-logged concentrations of IFNα-2a variants (1000 to 0.01 pM) in biological duplicates, and the mean fluorescence intensity (MFI) was measured after 5 days. EC_50_ values were calculated by a sigmoidal curve fitting with a constrained hill slope.

The influence of protein glyco-tagging on biological properties was evaluated by comparing the proinflammatory and antiproliferative capacities of modified IL-18 and IFNα-2a, respectively, with their unmodified counterparts (Figure 5A–C). IL-18 is a proinflammatory cytokine that facilitates type 1 immune responses. In the presence of IL-12, it stimulates IFN-γ production by CD4+ and CD8+ T cells as well as natural killer (NK) cells.^56^ Therefore, IFN-γ release was measured upon the stimulation of human PBMCs with IL-18 and its glyco-tagged variants (0.5 to 50 nM) in the presence of IL-12 (Figure 5A). Stimulation with glyco-tagged variants **6a** and **6b** resulted in comparable IFN-γ release as the nonmodified IL-18 (*p* = 0.054 for nonmodified IL-18 vs **6b**). The response to IL-18 followed a sigmoidal dose–response curve for PBMCs from two blood bank donors with an increase in maximal efficacy *E*_max_ by approximately 60 and 100% for **6a** and **6b**, respectively (Figure S15). To assess whether the natural high-affinity decoy receptor IL-18 BP, a secreted checkpoint inhibitor in cancer therapy,^56^ can interfere in IFN-γ release induced by IL-18 and its glyco-tagged variants, competition binding assays were conducted (Figure 5B). In fact, IFN-γ production stimulated by 10 nM of glyco-tagged IL-18 variants was overall diminished in the presence of IL-18 BP (0.5 and 10 nM), ranging from 35 to 90% at a concentration of 10 nM IL-18 BP. Overall, the data imply that modification by glyco-tagging does not compromise the biological activity of IL-18 variants compared to their unmodified counterpart.

IFNα-2a variants **7a** and **7b** were applied to CFSE (carboxyfluorescein succinimidyl ester)-labeled Daudi cells, and the impact of glyco-tagging on antiproliferative activity^49^ was evaluated by tracking diminished mean fluorescence intensities (MFI); i.e., a high MFI implies reduced proliferation and increased antiproliferative potency, and the dash line indicates MFI reduction of untreated cells (Figure 5C). It was found that purchased and heterologous expressed IFNα-2a (**7**) and the glyco-variant **7a** showed comparable antiproliferative potency (higher MFI values), whereas a higher concentration of glyco-variant **7b** was required to achieve the maximal antiproliferative capacity (EC_50_ = 12 vs 1.5 pM).

### Chemoenzymatic Synthesis of Glyco-Insulin Variants

Insulin is a pancreatic polypeptide hormone that is used for the treatment of diabetes. Its endogenous form produced by β-cells in response to glucose has a short half-life (∼5 min in human plasma) and is prone to oligomerization, aggregation, and fibrillization, which lead to decreased efficacy and potential adverse effects upon its application.^77,78^ Different approaches have been pursued to improve the properties of insulin, such as the use of additives and chemical modification. Peglispro, a PEG-modified form of insulin, was developed to provide more stable blood glucose levels over an extended period. However, due to the side effects caused by PEG, alternative methods such as glycosylation have been also explored.^79^ Several glyco-insulin variants have been synthesized employing various approaches, although they require demanding chemical synthesis or low-yielding conjugation approaches.^14,19^ In view of these earlier examples, we decided to test our tagging methodology for the synthesis of glyco-insulin using intact insulin from commercial sources as the starting material.

**Figure 6:**
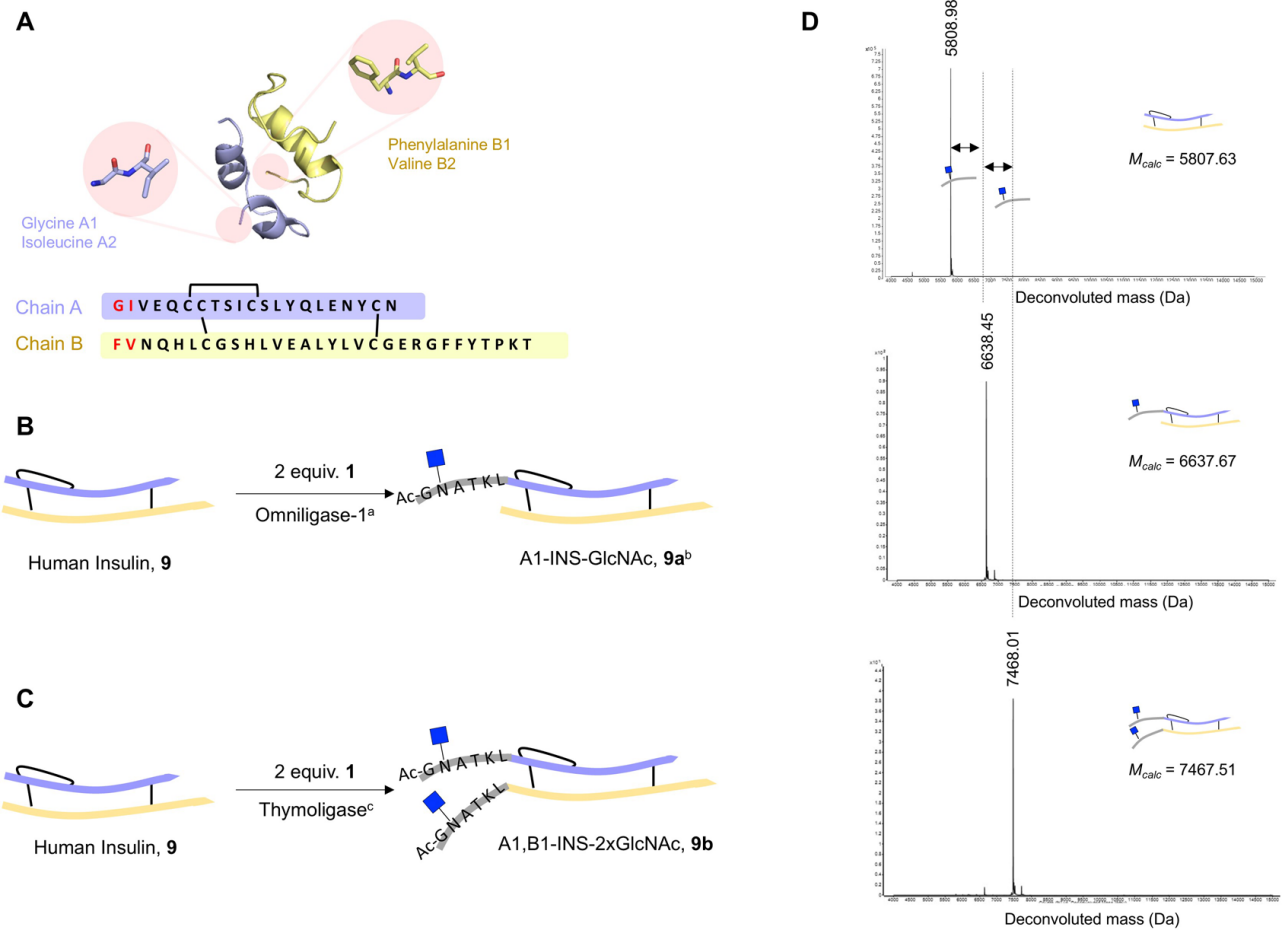
Peptidoligase-catalyzed human insulin-glycopeptide conjugation. (A) Structure (PDB file 1a7f) and amino acid sequence of native human insulin. Amino acid pairs at both chain A and B *N*-terminus are highlighted in red. (B, C) Chemoenzymatic synthesis of *N*-GlcNAc insulins **9a** and **9b**. Conversions were estimated by mass spectrometry analysis. ^a^Reaction conditions were 200 μM of **9**, 400 μM of **1,** 1.5 mol % omniligase-1, 100 mM tricine (pH 8.0), RT, and 2 h. A total of 56% of the starting material was converted into monoligated insulin. ^b^**9a** was obtained in 30% isolated yield. Selectivity of the reaction was determined by the treatment of the sample with DTT and detection of the individual peptides. ^c^Reaction conditions were 200 μM **9**, 400 μM **1**, 1.5 mol % thymoligase, 100 mM tricine (pH 8.0), and RT 2 h. If unmodified protein or monoligated insulin remained after 2 h, a second portion of glycopeptide **1** was added and allowed to react for an additional 2 h. Addition of two units of glycopeptide occurred in >95% conversion. **9b** was obtained in 40% isolated yield. (D) Deconvoluted mass spectra of intact human insulin **9** and glycoinsulin variants **9a** and **9b**.

Human insulin is composed of an A and B peptide chain (A: amino acids A1–A21, B: amino acids B1–B30) that are connected by two interchain disulfide bonds. The two *N*-terminal amino acids of the A chain are Gly-Ile and that of the B chain Phe-Val, which were both expected to be good substrates for peptidoligases (Figure 6A).^42^ Treatment of native human insulin (**9**) with 2 equiv of glycopeptide ester **1** in the presence of omniligase-1 resulted mainly in the formation of monoligated insulin as indicated by MS analysis (Figure 6B). The starting material and a small amount of bis-ligated insulin were also detected (56% conversion into monoligated insulin with an estimated selectivity of 86%). The use of a large excess of glycopeptide ester **1** (20 equiv, Figure S8 for more details) resulted in a larger conversion, but the monoligated insulin (INS-GlcNAc) was still the main product albeit with lower selectivity (70% conversion with 66% selectivity for the monoligated product). On the other hand, full conversion of insulin into bis-ligated insulin **9b** (A1,B1-INS-2xGlcNAc, Figure 6C) was achieved by using thymoligase in the presence of 2 equiv of **1** after a reaction time of 2 h as indicated by MS analysis. Thymoligase was developed to accept negatively charged amino acids (Asp) at position P1′^54^ and demonstrated superior activity for protein glyco-tagging compared with omniligase-1.

The mono- and bismodified glyco-insulins were purified by preparative reverse phase HPLC using a C18 column, and their identity and structural integrity were analyzed by ESI-QTOF mass spectrometry. Deconvoluted mass spectra of **9a** and **9b** displayed single peaks with *m*/*z* of 6637 and 7468 Da, respectively, corresponding to the addition of one and two GlcNAc-containing peptides (Figure 6D). The *m*/*z* peaks corresponding to individual A and/or B chains were not detected, supporting the compatibility of the methodology with the presence of disulfide bonds. The precise identity of monoligated insulin, compound **9a**, was ascertained by treatment with dithiothreitol (DTT) followed by MS analysis of the individual peptides. The *m*/*z* signals corresponding to glycopeptide addition to the A-chain and almost no modified B-chain were detected (Figure S9 for additional details), and the selectivity appears to be greater than >95%. These results indicate that the substrate specificity of omniligase-1 resembles that of its parent peptiligase, which prefers small amino acids in the S1′ pocket such as Gly, Ala, or Ser.^45,46^ In the case of thymoligase, the S1′ pocket was engineered to introduce an L217R point mutation that could promote favorable cation-π interactions. It is possible that this modification also facilitates the recognition of Phe at the *N*-terminus of the B-chain of insulin, thus providing full conversion to bis-ligated product **9b**.

**Figure 7:**
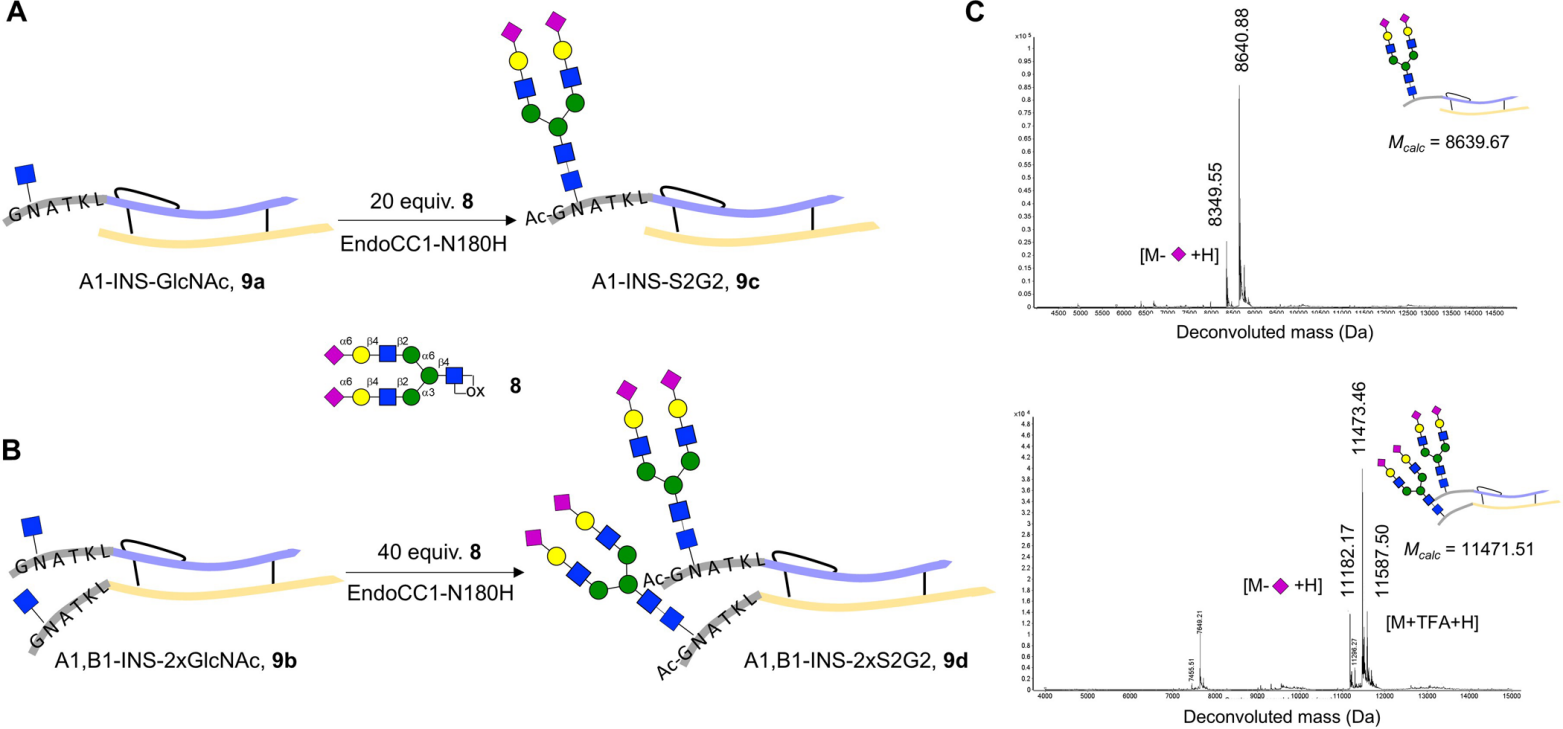
Chemoenzymatic synthesis of disialo-glycoinsulin analogues. (A, B) Chemoenzymatic installation of sialo complex-type biantennary oxazoline **8** into glycosylated insulins **9a** and **9b**. Conditions: 200 μM **9a/9b**, 20–40 equiv of **8**, 0.1 mol % EndoCC1-N180H, Tris-HCl 20 mM (pH 7.0), RT, 1 h. If the starting protein remained, a second portion of oxazoline **8** was added and allowed to react for an additional 1 h. Glycoinsulins **9c** and **9d** were obtained in 50 and 74% isolated yield, respectively. (C) Deconvoluted mass spectra of glycoinsulin variants **9c** and **9d**.

Next, attention was focused on the modification of GlcNAc moieties of **9a** and **9b** with complex sialylated *N*-glycan (Figure 7A,B). Thus, treatment of **9a** and **9b** with oxazoline **8** in the presence of EndoCC-N180H for 1 h resulted in the installation of complex-type glycans at the GlcNAc sites. Peaks with *m*/*z* of 8640.88 and 11473.46 Da matched the expected molecular weights for the addition of one or two complex glycans to afford **9c** and **9d,** respectively (Figure 7C). Also, minor peaks resulting from the loss of sialic acid and the formation of TFA adducts were detected, which most likely arise from in-source MS fragmentation or ion addition.^80^ The glyco-insulins were purified by HPLC using a C18 column to give homogeneous **9c** and **9d** in isolated yields of 50 and 74%, respectively. Thus, the two-step modification offers a highly convergent strategy to prepare well-defined glycosylated insulin variants from readily available native human insulin.

### Physical Properties of Glyco-Insulin Derivatives

In the pancreas, human insulin is stored in β-cells as an inactive and symmetric Zn^2+^ coordinated hexamer. In response to an increase in blood glucose levels, insulin is released to the bloodstream where it rapidly dissociates to form physiologically active monomers. Whereas the inactive hexamer is rather stable, the monomeric form is not and can partially unfold and then self-associate into oligomers, which are considered hallmarks of the prefibrillar phase that rapidly evolve to form larger aggregates and fibrils. Such aggregation compromises the therapeutic use of insulin.^81^

**Figure 8:**
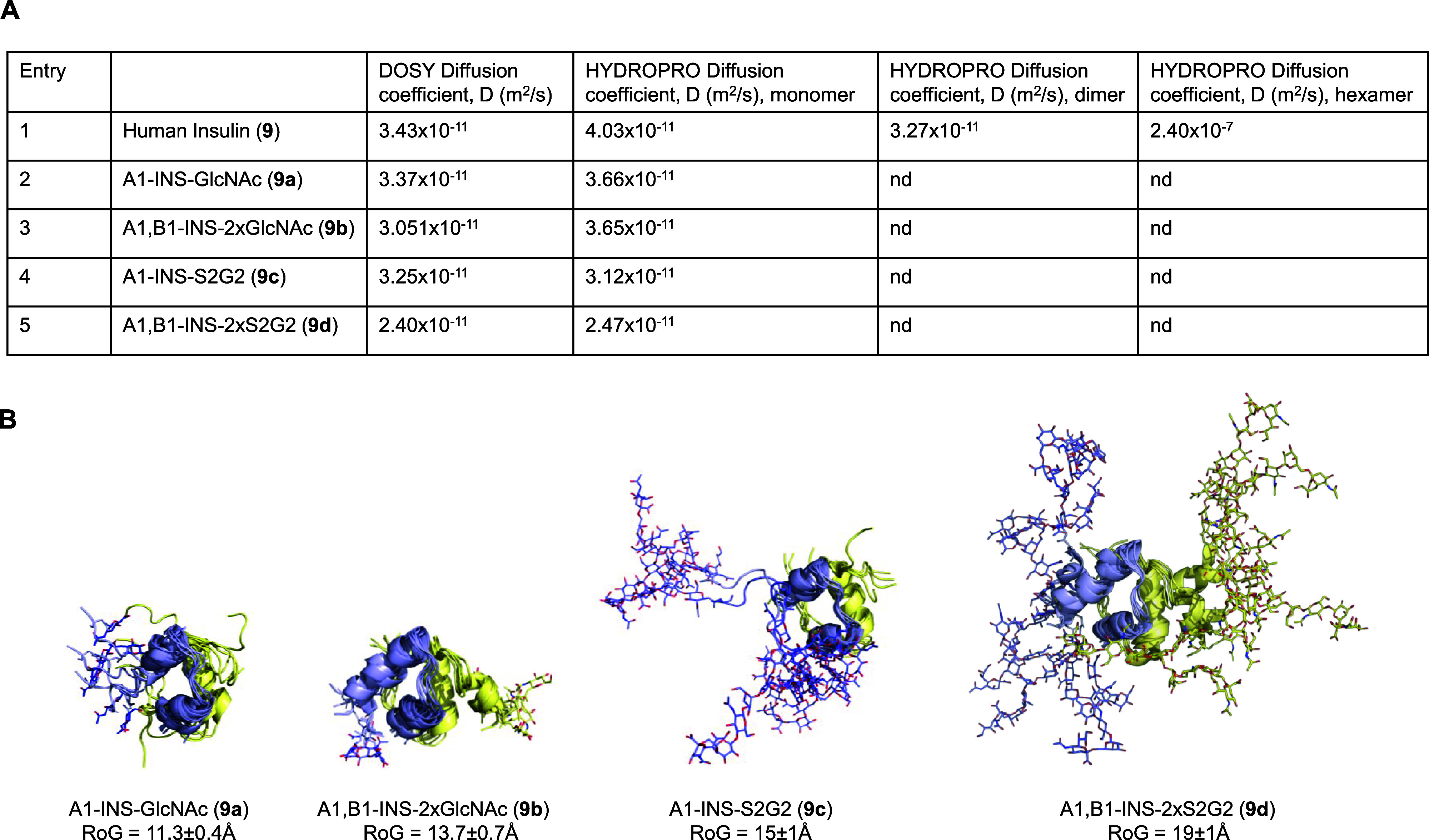
Analysis of the oligomeric state of human insulin **9** and derived glycoinsulins **9a**–**d**. (A) Diffusion coefficients of human insulin **9** and glycoinsulin variants **9a**–**d** determined by diffusion ordered spectroscopy NMR experiments (2D ^1^H-DOSY-NMR). Calculated diffusion coefficients of human insulin monomer, dimer, and hexamer as well as those for glycoinsulins **9a**–**d** monomers are also indicated. (B) 3D structural models of glycoinsulins **9a**–**d**. The superimposition of several MD snapshots showed the increased sampled conformational space of disialoglycosylated insulins and the impact on the radius of gyration of the molecule.

Diffusion ordered NMR spectroscopy (DOSY) can provide information about the size/shape of molecules.^82^ Therefore, we used this experimental approach to investigate the oligomeric state of the glyco-insulin variants in solution.^83^ First, the insulin derivatives were investigated at low pH and low salt concentration because these conditions are known to greatly reduce aggregation. Thus, 50 μM buffered solutions of human insulin **9** and the different glyco-insulins **9a**–**d** at pH 1.6 were prepared, and 2D ^1^H-DOSY-NMR experiments were acquired to provide diffusion coefficients D (Figure 8A). All DOSY spectra displayed a single set of NMR signals (Figure S14), indicating that human insulin **9** and its glycovariants **9a**–**d** are present in a single oligomeric state, or if they exist as an ensemble of different oligomeric forms, they are in fast exchange.

To discern the oligomeric state of human insulin under the employed experimental conditions, theoretical diffusion coefficients were calculated using the simulation software HYDROPRO and compared to the experimental values. X-ray crystal structures of human insulin monomer, dimer, and hexamer (PDB ID 3aiy) were used as coordinate input files. The experimental diffusion coefficient of bovine pancreatic ribonuclease A, a small globular protein of 13.7 kDa (PDB ID 3rn3), was used as a reference to standardize the computed values. In agreement with previously reported values, the determined diffusion coefficient for unmodified human insulin **9** matched a dimer as the main species (Figure 8A, entry 1).^84^ In particular, a 4:1 ratio in favor of insulin dimer would account for the measured diffusion coefficient when monomer and dimer species were considered to calculate the weighted mean value. For comparison purposes, 3D structural models of **9a**–**d** monomers were built using AlphaFold in combination with the glycoprotein builder module implemented in GLYCAM-Web portal, and their dynamic behavior was interrogated by molecular dynamics (MD) simulations (Figure 8B). Installation of GlcNAc or S2G2-bearing glycopeptides increased the radius of gyration (RoG) and, therefore, the hydrodynamic radius of the analyzed proteins. Predicted RoG and diffusion coefficients for disialoglycoinsulins **9c** and **9d**, carrying highly hydrophilic and conformationally flexible *N*-glycans, were substantially higher. Interestingly, experimentally determined diffusion coefficients for **9a**–**9d** were in close agreement to the calculated values of the monomeric species (Figure 8A, entries 2–5). Thus, based on their diffusion properties, peptide glyco-tagging and especially the introduction of large *N*-glycans, such as in the case of glycoinsulins **9c** and **9d**, shift the oligomeric equilibrium of human insulin toward monomers.

**Figure 9:**
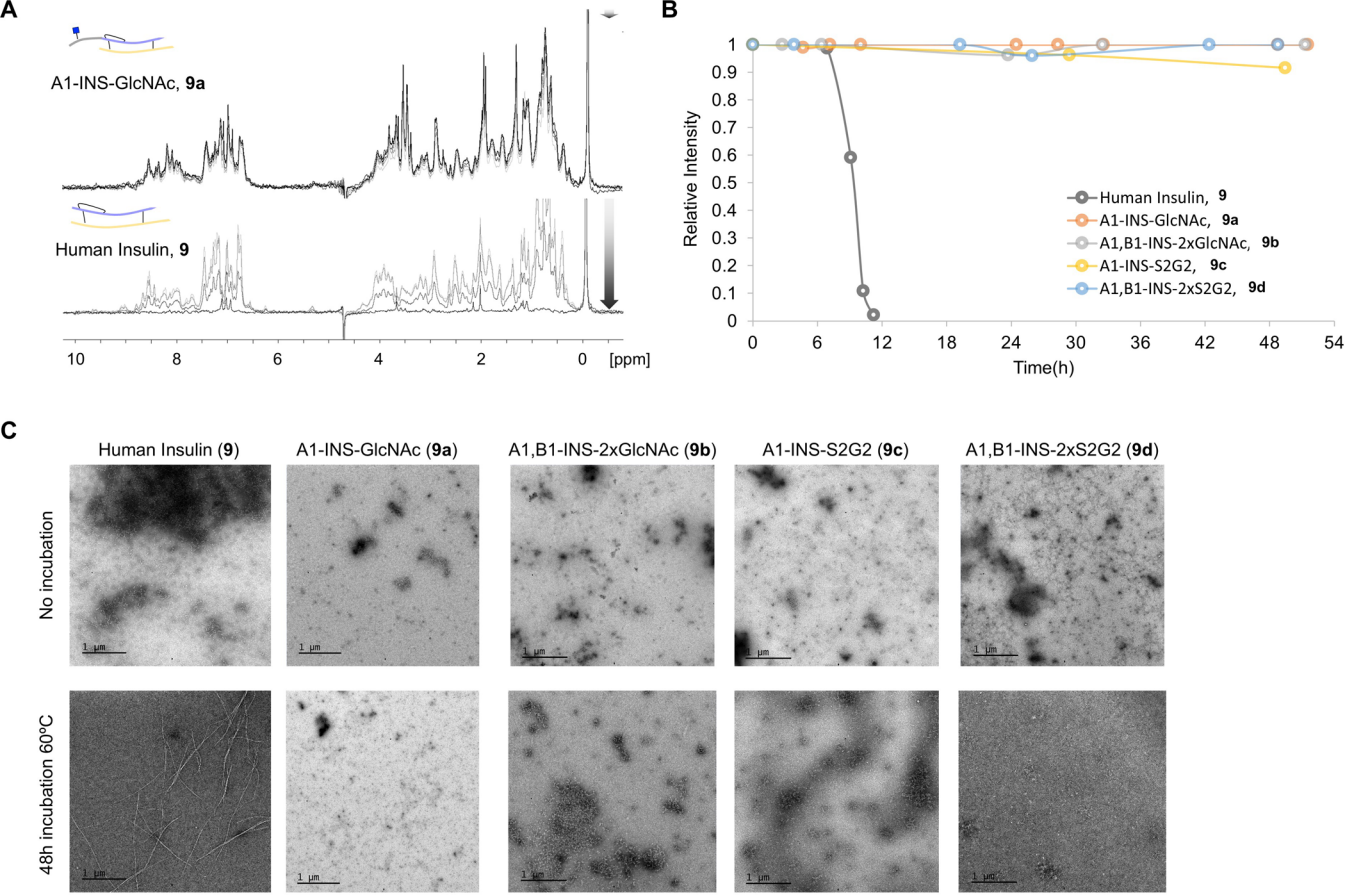
Aggregation properties of human insulin **9** and glycoinsulin variants **9a**–**d**. (A) ^1^H NMR monitoring of signal intensity changes for human insulin **9** and glycosylated insulin **9a** over sample incubation at 60 °C. A similar trend was observed for synthesized insulins **9b**–**d**. (B) Decay of relative intensity of protein signals over time. Samples (50 μM) in phosphate buffer pH 1.6 containing 150 mM NaCl were analyzed. (C) TEM images of 30 μM insulin **9** and glycoinsulin **9a**–**d** samples before and after 48 h of incubation at 60 °C.

To investigate the amyloidogenic properties of the glyco-tagged insulin variants, experimental conditions that favor aggregation were applied.^85^ Thus, 50 μM protein samples in buffered solutions at pH 1.6 and containing 150 mM NaCl were incubated at 60 °C, and their aggregation kinetics were analyzed by NMR. In the case of human insulin **9**, acquisition of ^1^H NMR experiments allowed the detection of the decay of the ^1^H signal intensities over time. After 12 h of incubation, the signal intensity was reduced to 2% (Figure 9A,B). Close monitoring allowed the identification of sigmoidal kinetics, which comprised a typical lag phase followed by a faster growth/aggregation step. No new peaks appeared. These observations are indicative of the conversion of small oligomers (NMR visible species) into large oligomeric species whose NMR signals are broadened and not NMR-visible anymore. Remarkably, almost no aggregation of glycoinsulin variants **9a**–**d** was observed, and the decay in signal intensity was less than 10% after a long incubation time of 49 h (for A1-INS-S2G2, **9c**) (Figure 9A,B).

Transmission electron microscopy (TEM) was employed to further analyze the possible aggregation of insulin and insulin derivatives after an incubation period of 48 h at 60 °C. As expected, only fibril formation was observed for human insulin **9**, whereas large structures were absent for the glycoinsulins **9a**–**d** (Figure 9C). Collectively, the results demonstrate that glycosylation protects insulin from aggregation. Although the mechanism underlying insulin aggregation and fibrillization is not fully understood, it is widely accepted that hydrophobic interactions facilitate the unfolding and misfolding of the polypeptide chains resulting in agglomeration, fibrillogenesis, and precipitation.^86,87^ The introduction of hydrophilic entities such as glycans may disrupt unfavorable hydrophobic interactions.

### Biological Activities of Glycoinsulin Variants

**Figure 10:**
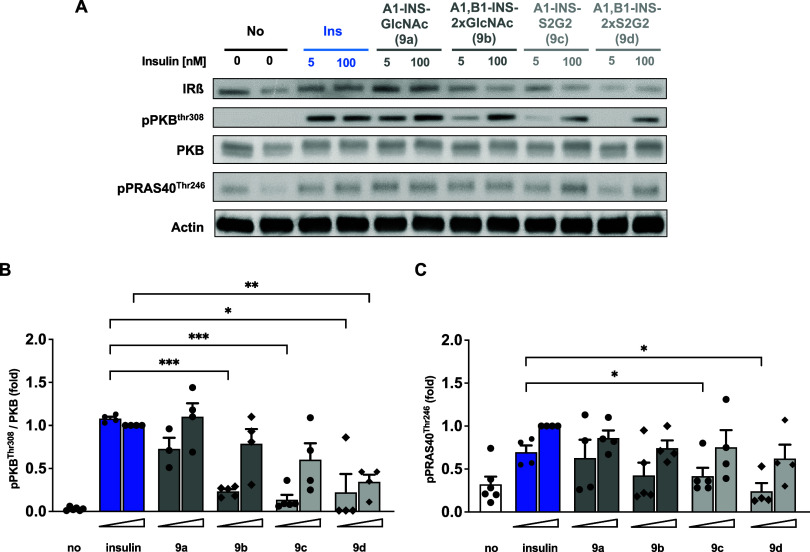
Insulin signaling by human insulin and glycoinsulin variants **9a**–**d**. SGBS human adipocyte cells (*n* = 4) were differentiated for 14 days in adipogenic medium and serum-starved for 16 h before stimulation for 5 min with human insulin and the glycovariants **9a**–**d** at 5 and 100 nM. The cells were lysed, and phosphorylation of PKB at residue Thr308 and PRAS at residue Thr246 was detected by Western blot. Phosphorylation levels were quantified for the phosphorylation sites. (B) Thr308 of PKB and (C) Thr246 of the substrate PRAS40 and normalized against the nonphosphorylated protein. Data are represented as means ± the standard error of the mean (SEM). Statistical significance was tested with a mixed effect model using restricted maximum likelihood (REML) for data fitting (implemented in Prism 10).

Binding of insulin to its cell surface receptor on metabolic cells triggers a complex cascade of intracellular events, inducing, among other events, glucose uptake by the translocation of glucose transporter type 4 (GLUT4).^88^ A central event of this signaling cascade is the phosphorylation of protein kinase B (PKB) on its Thr308 residue (by PDK1) and Ser473 (by mTORC2), resulting in kinase activation and subsequent phosphorylation of multiple downstream targets, such as PRAS40, an inhibitory binding partner for mammalian target of rapamycin (mTOR).^89^ Activated mTOR regulates upstream a plethora of cell-specific biological processes, such as glucose metabolism, lysosomal biogenesis, and lipid synthesis. To assess the impact of insulin glycosylation on downstream signaling, fully differentiated Simpson–Golabi–Behmel syndrome (SGBS) human adipocyte cells were stimulated with unmodified and the glyco-tagged insulin variants **9a**–**d** at 5 and 100 nM for 5 min. Thr308- and Ser473-PKB phosphorylation as well as phosphorylation of the Thr246-PRAS40 substrate was quantified by Western blotting (Figure 10A and Figure S16A). The sensitivity of the test system was assessed by the application of all variants at a concentration of 5 nM. All variants were able to induce phosphorylation at Thr308- and Ser473-PKB; however, different levels of phosphorylation were observed for the various glyco-variants, in particular at a low concentration of 5 nM (Figure 10B). Introduction of single GlcNAc moieties at the A-chain or both chains (**9a** and **9b,** respectively) had only a marginal effect on the phosphorylation levels when compared to that of unmodified insulin. In contrast, the installation of large complex glycans, especially in the case of variant **9d**, resulted in reduced phosphorylation. Further downstream, a similar behavior was observed by quantifying the phosphorylation levels of the mTOR inhibitory substrate PRAS40 (Figure 10C) and the phosphorylation of various other PKB substrates using an antibody recognizing its consensus motif for phosphorylation (Figure S16B).

## Conclusions

A chemoenzymatic methodology for glyco-tagging of native proteins is described that exploits peptidoligases to fuse a peptide ester carrying an *N*-acetyl-glucosamine (GlcNAc) moiety to the *N*-terminus of a protein, generating an *N*-GlcNAc modified glycoprotein. The GlcNAc moiety of the resulting glycoprotein can be elaborated into complex glycans by *trans*-glycosylation using well-defined sugar oxazolines and mutant forms of endo β-*N*-acetylglucosaminidases (ENGases). The workflow is orthogonal with high yielding protein expression in *E. coli* that cannot glycosylate proteins. The strategy was employed for the preparation of glycosylated variants of several proteins, including cytokines IL-18 and IFNα-2a, and human insulin. It was found that the glycovariants maintained the protein functionality. In the case of human insulin, the new constructs displayed improved physicochemical properties. A modification in human insulin may result in unwanted antigenic responses; for example, porcine insulin, which differs from human insulin by one amino acid (position 30 of the B-chain), induced antibodies in a small number of patients.^90^ To prevent such responses, we selected a highly conserved *N*-glycosylation sequon. Furthermore, it is expected that the glycan moiety will shield the newly introduced peptide epitope from recognition by B-cell receptors, thereby preventing antigenic responses. Future studies should focus on examination of *in vivo* pharmacokinetic (PK) properties of the modified proteins. Such studies can also examine the anticipated lack of antigenic properties of the newly introduced glycotags.

Engineered mutants of peptidoligases have been described that have different activities for the *N*-terminal sequences,^42^ which offer the prospect of modifying many different native proteins by glycotags. Additionally, chemoenzymatic methodologies have been described that make it possible to prepare large panels of *N*-linked glycans starting from a readily available biantennary glycopeptide isolated from egg yolk powder that can be converted into multiantennary *N*-glycans by using recombinant glycosyl transferases and modified sugar nucleotide donors.^91^ In addition to *trans*-glycosylations, it is the expectation that the GlcNAc moiety of the neo-glycoproteins can be stepwise extended by glycosyl transferases to provide other glycoforms.^92^ The combination of these methodologies paves the way to modify native proteins in a well-defined manner with a diverse range of glycans for structure–function *in vitro* and *in vivo* studies, and it is expected to provide a much needed tool for improving properties of therapeutic proteins. In addition to improving PK properties, the glyco-tagging approach can also be employed for the attachment of functional glycans to facilitate tissue targeting and cellular uptake. For example, mannose-6-phosphate (M6P) mediates cellular uptake and lysosomal targeting of proteins having this modification. Cell engineering strategies have been employed to introduce M6P into therapeutic proteins for enzyme replacement therapy.^93^ However, such an approach can also introduce unwanted glycans, such as high mannosides that increase clearance rates. In addition, controlling glycosylation with M6P during manufacturing has been proven difficult.^93,94^ Chemical approaches have been used to modify therapeutic proteins with M6P, such as in the case of recombinant acid alpha-glucosidase (Gaa), that was modified with bis-phosphorylated oligosaccharide.^95^ The resulting neo-glycoconjugate (avalglucosidase alfa) exhibits improved cellular uptake and better targets muscle cells, resulting in greater glycogen reduction in *Gaa* KO mice. Avalglucosidase alfa has been examined in clinal trials and approved for the treatment of late onset Pompe disease (brand name, Nexviazyme). Avalglucosidase alfa is produced by a random modification with M6P by periodate-mediated oxidation of sialosides followed by hydrazone ligation.^95^ Oxazolines of M6P-containing glycans have been described,^96^ and thus, it is the expectation that the glyco-tagging approach can be employed to prepare well-defined therapeutic proteins having an M6P moiety.

We also expect that the glyco-tagging approach will find applications in vaccine development. For example, modification of an antigen with a ligand for C-type lectins such as dendritic cell specific ICAM grabbing nonintegrin (DC-SIGN) or Langerin will result in targeting and increased uptake by dendritic cells (DC) resulting in improved presentation of T-cellepitopes.^97^ The glyco-tagging approach will make it possible to install glycans such as Lewis^y^, which is a ligand for DC-SIGN, in peptide- or protein based vaccines.

## Supporting Information Available

Methods, analytical data, and additional figures (PDF)
